# Raloxifene Ameliorates Glucosamine-Induced Insulin Resistance in Ovariectomized Rats

**DOI:** 10.3390/biomedicines9091114

**Published:** 2021-08-30

**Authors:** Chung-Hwan Chen, Tsung-Lin Cheng, Chi-Fen Chang, Hsuan-Ti Huang, Sung-Yen Lin, Meng-Hsing Wu, Lin Kang

**Affiliations:** 1 Orthopaedic Research Center, College of Medicine, Kaohsiung Medical University, Kaohsiung 80701, Taiwan; hwan@kmu.edu.tw (C.-H.C.); junglecc@kmu.edu.tw (T.-L.C.); hthuang@kmu.edu.tw (H.-T.H.); sungyenlin@kmu.edu.tw (S.-Y.L.); 2 Department of Orthopedics, Kaohsiung Medical University Hospital, Kaohsiung Medical University, Kaohsiung 80701, Taiwan; 3 Regeneration Medicine and Cell Therapy Research Center, Kaohsiung Medical University, Kaohsiung 80701, Taiwan; 4 Departments of Orthopedics, College of Medicine, Kaohsiung Medical University, Kaohsiung 80701, Taiwan; 5 Department of Orthopedics, Kaohsiung Municipal Ta-Tung Hospital, Kaohsiung Medical University, Kaohsiung 80145, Taiwan; 6 Department of Healthcare Administration and Medical Informatics, Kaohsiung Medical University, Kaohsiung 80701, Taiwan; 7 Institute of Medical Science and Technology, National Sun Yat-Sen University, Kaohsiung 80420, Taiwan; 8 Graduate Institute of Animal Vaccine Technology, College of Veterinary Medicine, National Pingtung University of Science and Technology, Pingtung 912301, Taiwan; 9 Graduate Institute of Materials Engineering, College of Engineering, National Pingtung University of Science and Technology, Pingtung 912301, Taiwan; 10 Department of Physiology, College of Medicine, Kaohsiung Medical University, Kaohsiung 80701, Taiwan; 11 Department of Anatomy, School of Medicine, China Medical University, Taichung 40402, Taiwan; cfchang@mail.cmu.edu.tw; 12 Department of Obstetrics & Gynecology, College of Medicine, National Cheng Kung University, Tainan 70101, Taiwan; mhwu68@mail.ncku.edu.tw; 13 Department of Obstetrics and Gynecology, National Cheng Kung University Hospital, College of Medicine, National Cheng Kung University, Tainan 70101, Taiwan

**Keywords:** apoptosis, endoplasmic reticulum stress, glucosamine, pancreatic β-cell dysfunction, ovariectomy, raloxifene

## Abstract

Osteoarthritis (OA) and osteoporosis (OP) are common among older women, especially postmenopausal women. Glucosamine (GlcN) is a common medication for OA, but it may induce insulin resistance and β-cell dysfunction, especially if ovarian hormones are lacking. Raloxifene (RLX) is a selective estrogen receptor modulator and also an OP drug. Previously, we found that estrogen could improve GlcN-induced insulin resistance in ovariectomized (OVX) rats. Here, we further hypothesized that RLX, similarly to estrogen, can ameliorate GlcN-induced insulin resistance in OVX rats. We used GlcN to induce insulin resistance in OVX rats as a model for evaluating the protective effects of RLX in vivo. We used a pancreatic β-cell line, MIN-6, to study the mechanisms underlying the effect of RLX in GlcN-induced β-cell dysfunction in vitro. Increases in fasting plasma glucose, insulin, and homeostasis model assessments of insulin resistance in OVX Sprague Dawley rats treated with GlcN were reversed by RLX treatment (*n* = 8 in each group). Skeletal muscle GLUT-4 increased, liver PEPCK decreased, pancreatic islet hypertrophy, and β-cell apoptosis in OVX rats treated with GlcN was ameliorated by RLX. The negative effects of GlcN on insulin secretion and cell viability in MIN-6 cells were related to the upregulation of reticulum (ER) stress-associated proteins (C/EBP homologous protein, phospho-extracellular signal-regulated kinase, phospho-c-JunN-terminal kinase), the expression of which was reduced by RLX. Pretreatment with estrogen receptor antagonists reversed the protective effects of RLX. GlcN can induce insulin resistance, β-cell dysfunction, and apoptosis in OVX rats and increase ER stress-related proteins in β-cells, whereas RLX can reverse these adverse effects. The effects of RLX act mainly through estrogen receptor α; therefore, RLX may be a candidate drug for postmenopausal women with OA and OP.

## 1. Introduction

Metabolic syndrome and type 2 diabetes mellitus (T2DM) are prevalent health problems [1]. Insulin resistance (IR) is a hallmark of T2DM and metabolic syndrome, which have higher prevalence in postmenopausal women than in premenopausal women. Osteoarthritis (OA) is also prevalent in postmenopausal women [2]. T2DM, with its related chronic hyperglycemia and IR, induces possibly pathogenic effects in OA through oxidative stress and chronic low-grade inflammation [3]. Glucosamine (GlcN) is a nutritional supplement widely used for OA [4,5]. However, studies have reported that GlcN affects glucose tolerance and IR [3,6]. GlcN inhibits the insulin production of pancreatic β-cells [7,8]. The implications of clinical data regarding GlcN and glucose metabolism are conflicting. Some clinical studies have observed harmful effects of GlcN on glucose metabolism [9,10], whereas others have reported no effects [11,12].

Ovarian estrogen, 17 β-estradiol (E_2_), has ameliorated both insulin sensitivity and insulin production in animal and human studies [13,14]. The risk of T2DM increased after ovariectomy in an animal study, whereas estrogen application ameliorated T2DM and enhanced insulin sensitivity [15]. In pancreatic β-cells, E_2_ can ameliorate glucolipotoxicity, oxidative stress, and apoptosis [14,16]. Estrogen receptors can regulate the function and survival of β-cells [17], although estrogen use is associated with an increased risk of various cancers [18].

Selective estrogen receptor modulators (SERMs) act on estrogen receptors with agonist or antagonist activity, depending on the tissue type. Raloxifene (RLX) is an SERM used for the treatment of postmenopausal osteoporosis (OP) because of its activity as an estrogen receptor agonist in bone [19,20,21,22,23,24,25,26,27,28]. The effect of RLX can be tissue- or species-specific [29]. One study using a pancreatic β-cell line (INS-1 cells) demonstrated that RLX behaves as both an estrogen receptor antagonist through nuclear estrogen response element-dependent actions and as an estrogen receptor agonist by suppressing triglyceride accumulation [30]. The endoplasmic reticulum (ER) regulates intracellular calcium concentrations and protein folding and trafficking [31,32,33,34]. ER stress, the disruption of these ER functions, is related to T2DM in humans [35,36,37]. ER stress can be induced by GlcN and cause cell death [38,39]. In our previous study, we demonstrated that GlcN-induced IR in ovariectomized (OVX) rats was related to increased pancreatic islet size [40]. We also observed the protective effects of E_2_ in GlcN-induced pancreatic β-cell dysfunction [41]. The rescue effects of RLX in GlcN-induced IR and pancreatic β-cell dysfunction have not been reported. In the current study, we used OVX rats treated with GlcN to induce IR and studied the protective effects of RLX in vivo. We also used pancreatic β-cell lines, MIN-6 cells, to study the protection and underlying mechanisms of RLX in GlcN-induced β-cell dysfunction in vitro.

## 2. Materials and Methods

### 2.1. Ethics Statement

All procedures were performed in accordance with the Institutional Guidelines for Animal Care of National Cheng Kung University, the Use of Laboratory Animals of the National Institutes of Health, and the guidelines of the Animal Welfare Act.

### 2.2. Experimental Animals

Twelve-week-old Female Sprague Dawley rats were purchased from the Animal Center of National Cheng Kung University Medical College and housed under standard laboratory conditions with free access to food and water. After acclimation, the rats were randomly allocated to one of 5 treatments: (1) sham operation (Sham group); (2) sham with 750 mg/kg/d GlcN (Sigma-Aldrich, St. Louis, MO, USA) intraperitoneally (ip) injected for 14 days (Sham + GlcN group); (3) ovariectomy (OVX group); (4) ovariectomy with 750 mg/kg/d GlcN ip treated for 14 days (OVX + GlcN group); or (5) ovariectomy with 750 mg/kg/d GlcN ip treated for 14 days with subcutaneous RLX at 0.5 mg/kg/d (Sigma Chemical Co., St. Louis, MO, USA; OVX + GlcN + RLX group; *n*  =  8 in each group). Surgeries were performed under ip administered sodium pentobarbital (Sigma-Aldrich) anesthesia through bilateral lower back skin incisions [40,41,42,43,44,45]. Twelve weeks after the surgery, GlcN was administered for two weeks [40,41,45].

### 2.3. Intraperitoneal Glucose Tolerance Test

An intraperitoneal glucose tolerance test (IPGTT) was administered with the rats fasted for 6 h after all of the treatments were completed. Blood samples for the measurement of plasma glucose and insulin were drawn from the femoral vein before glucose loading (1 mg/kg, ip) at baseline (time 0). Blood samples were obtained at 30, 60, 90, and 120 min after glucose loading [40,41,45].

### 2.4. Plasma Glucose and Insulin Concentrations

Plasma glucose levels were evaluated by a commercial kit reagent for glucose (Biosystems SS, Barcelona, Spain) by an analyzer (Quik-Lab, Elkhart, IN, USA). Insulin concentration was evaluated by an insulin enzyme-linked immunosorbent assay (ELISA) kit (Mercodia AB, Uppsala, Sweden), as described previously [40,45].

### 2.5. Determination of IR in Rats

IR and β-cell function were assayed using homeostasis model assessments of IR (HOMA-IR). The glucose-insulin index and clinical HOMA-IR were determined to evaluate IR and compare groups after the concentrations of plasma glucose and insulin were measured. The glucose-insulin index was calculated as the product of the glucose and insulin areas under the curve (AUCs). HOMA-IR  =  [fasting glucose (mmol/L)]  ×  [fasting insulin (pmol/mL)]/22.5 [40,45].

### 2.6. Measurement of Islet Size

All pancreases were immersed in phosphate-buffered saline (PBS) containing 10% formaldehyde (*v*/*v*) and kept at 4 °C for 2 days. After dehydration, the specimens were fixed in paraffin. The specimens were sliced into 5 μm thick sections with 50 μm distances; then, hematoxylin and eosin staining was performed. For each staining, more than three serial sections were used. The area of each islet was decided by Image-Pro Plus (Media Cybernetics, Inc., Rockville, MD, USA) with a total of 210–230 islets in each section [40,45].

### 2.7. Immunofluorescence Stains for Insulin and Terminal Deoxynucleotidyl Transferase dUTP Nick End-Labeling in Pancreatic Islets

Sequential 5 μm thick pancreas sections around 2  ×  1 cm^2^ were immunostained for transferase dUTP nick end-labeling (TUNEL), insulin, and DAPI. The immunofluorescence staining intensity was quantified using Image-Pro Plus (Media Cybernetics, Inc., Rockville, MD, USA) [41,46,47,48].

### 2.8. Western Blot Analysis for PEPCK in the Liver and GLUT-4 in the Soleus Muscle

The livers and soleus muscles were harvested immediately after the rats were killed, as described previously [40,45]. In brief, the tissues were washed with cold PBS and cut into 200–300 mg portions. After homogenization of the liver and soleus muscle, the homogenates (50 µg) were separated through sodium dodecyl sulfide–polyacrylamide gel electrophoresis, and Western blot analysis was performed using either an anti-rat glucose transport protein subtype 4 (GLUT-4) antibody (R&D system, Inc., Minneapolis, MN, USA) (1:1000) in the soleus muscle or an anti-rat phosphoenolpyruvate carboxykinase (PEPCK) antibody (R&D system, Inc., Minneapolis, MN, USA) (1:1000) in liver tissue.

### 2.9. Cell Culture and Compound Stimulation

MIN-6 cells were kept in a monolayer culture at 37 °C and 5% (*v*/*v*) CO_2_ in Dulbecco’s modified Eagle’s medium (DMEM) supplemented with 10% (*v*/*v*) fetal bovine serum (FBS), 1.0 × 10^5^ U/L penicillin, and 100 g/L streptomycin. MIN-6 cells were incubated with RLX 1 at μmol/L (Sigma Chemical Co.) [30], ICI 182,780 (antagonist to both estrogen receptor α and estrogen receptor β, Tocris, Ballwin, MO, USA) at 1 μmol/L, and methyl-piperidino-pyrazole (MPP, antagonist specific to estrogen receptor α, Tocris Cookson, Ellisville, MO, USA) at 1 μmol/L, as indicated, for 72 h. After estrogen receptor ligand treatment, the cells were treated with GlcN (Sigma Chemical Co.) at 10 mmol/L for 6 h before assessment in the following experiments.

### 2.10. Extracellular Insulin Levels

The MIN-6 cells were incubated in 6-well plates (1.0 × 10^4^ cells/well) with 1 μmol/L ICI 182,780 or 1 μmol/L MMP for 1 h in high-glucose (4.5 g/L) DMEM if needed, and then exposed to 1 μmol/L RLX for 24 h. The cells were transferred to low-glucose (1 g/L) DMEM with 1 μmol/L RLX for another 24 h. After the supernatant was removed, the cells were washed twice with PBS and treated with 10 mmol/L GlcN for half an hour, and then exposed to glucose (5.5 mmol/L) for another half an hour. The insulin in the supernatant was assayed by an insulin ELISA kit (Mercodia AB, Uppsala, Sweden).

### 2.11. Cell Viability Analysis

MIN-6 cells were plated in 96-well plates (1.0 × 10^4^ cells/well). The effects of RLX on the viability of GlcN -treated MIN-6 cells were evaluated by MTT assay. After a 1-day culture, the cells were treated with RLX, GlcN, ICI 182,780, and MPP as mentioned, and an MTT solution was subsequently used. The precipitates were dissolved in DMSO, and the absorbance was evaluated by an ELISA reader (Thermo Molecular Devices Co., Union City, CA, USA) at 570 nm after a 4 h culture. The cell viability ratio was calculated as follows:

Inhibitory ratio (%)  =  [(OD control − OD treated)/OD control] × 100

### 2.12. Western Blot Analysis for Protein Expression Related to ER Stress

MIN-6 cells were treated as described in the preceding sections, and then proteins were harvested from the cell lysates after being treated with lysis buffer. We used 10% (*w*/*v*) sodium dodecyl sulfate–polyacrylamide gel electrophoresis to separate the protein lysates (50 µg). Western blot analysis was performed by antibodies against C/EBP homologous protein (CHOP), phospho-extracellular signal-regulated kinase (p-ERK), phospho-c-JunN-terminal kinase (p-JNK), and β-actin antibodies (Santa Cruz Biotechnology, Santa Cruz, CA, USA). The blots were treated with secondary antibodies. After washing, the blots were developed using the ECL Western blotting system (R&D system, Inc., Minneapolis, MN, USA) and quantified through laser densitometry.

### 2.13. Statistical Analysis

The in vivo data are expressed as the mean  ±  standard error of mean (SEM) for the number (*n*) of animals in each group as indicated in the Methods. Each in vitro experiment was repeated (*n*) 3 or more times, and the data are expressed as mean  ±  SEM. Statistical differences among groups were determined using the Friedman test in IPGTT and one-way analysis of variance in GLUT-4, *PEPCK*, TUNEL and the sizes of pancreatic islets. Dunnett range post hoc comparisons were used to determine the source of significant differences where appropriate [40,45]. A *p* value < 0.05 was considered significant.

## 3. Results

### 3.1. RLX Ameliorated Fasting Glucose, Insulin, and HOMA-IR in the OVX + GlcN Rats

**Figure 1:**
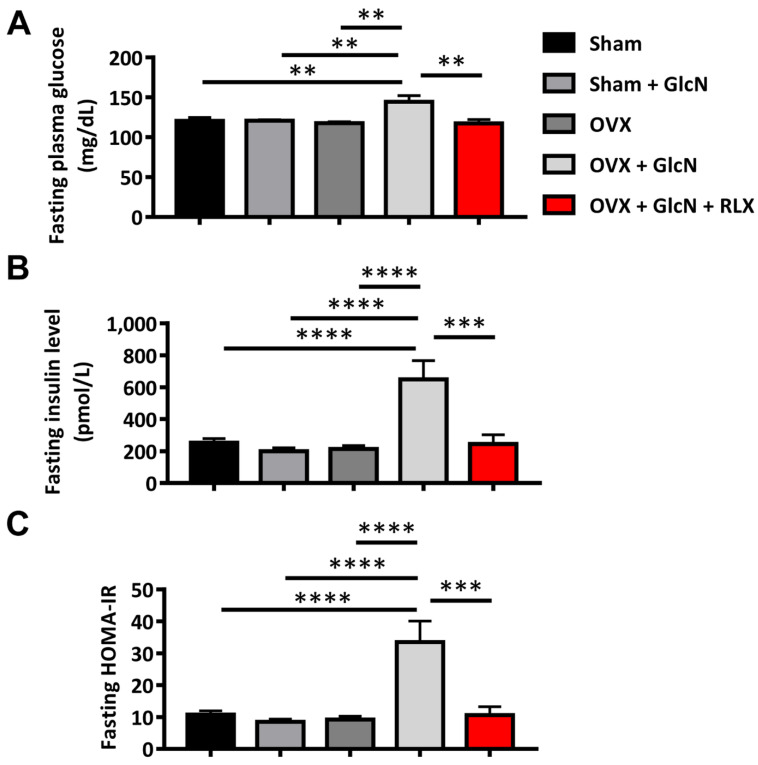
Levels of fasting glucose, insulin, and HOMA-IR (at time 0). The rats were randomly allocated to 5 treatments: (1) sham operation (Sham group), (2) sham with 750 mg/kg/d GlcN intraperitoneally (ip) injected for 14 days (Sham + GlcN group), (3) ovariectomy (OVX group), (4) ovariectomy with 750 mg/kg/d GlcN ip injected for 14 days (OVX+GlcN group), or (5) ovariectomy with 750 mg/kg/d GlcN ip injected for 14 days with subcutaneous RLX at 0.5 mg/kg/d. (*n* = 8 in each group). (**A**) The OVX + GlcN group exhibited higher fasting glucose than that in all other groups. RLX can significantly reduce fasting glucose levels in OVX rats. (**B**) The OVX + GlcN group exhibited higher fasting plasma insulin than that in all other groups. (**C**) RLX can significantly reduce fasting HOMA-IR in GlcN-treated OVX rats. ** *p* < 0.01; *** *p* < 0.001; **** *p* < 0.0001.

The OVX + GlcN group (144  ± 7.8 mg/dL) presented higher fasting glucose levels than that in the Sham (120 ± 4.2 mg/dL), Sham + GlcN (121 ± 1.2 mg/dL), OVX (117 ± 1.7 mg/dL), and OVX + GlcN + RLX (117 ± 4.9 mg/dL) groups (*p* < 0.01). RLX significantly reduced fasting glucose level in the OVX rats (Figure 1A). In addition, the OVX + GlcN group (649 ± 117 pmol/L) exhibited higher fasting plasma insulin level than that in all other groups (*p* < 0.001; Figure 1B). The OVX + GlcN group also exhibited higher fasting HOMA-IR (33.5 ± 6.6) than that in all other groups (*p* < 0.001; Figure 1C). RLX significantly reduced fasting plasma glucose, insulin levels (245 ± 57 pmol/L), and HOMA-IR (10.6 ± 2.6) in the OVX + GlcN rats.

### 3.2. RLX Ameliorated Insulin, Glucose, Glucose-Insulin Index, and HOMA-IR in Plasma during IPGTT in the OVX + GlcN Rats

**Figure 2:**
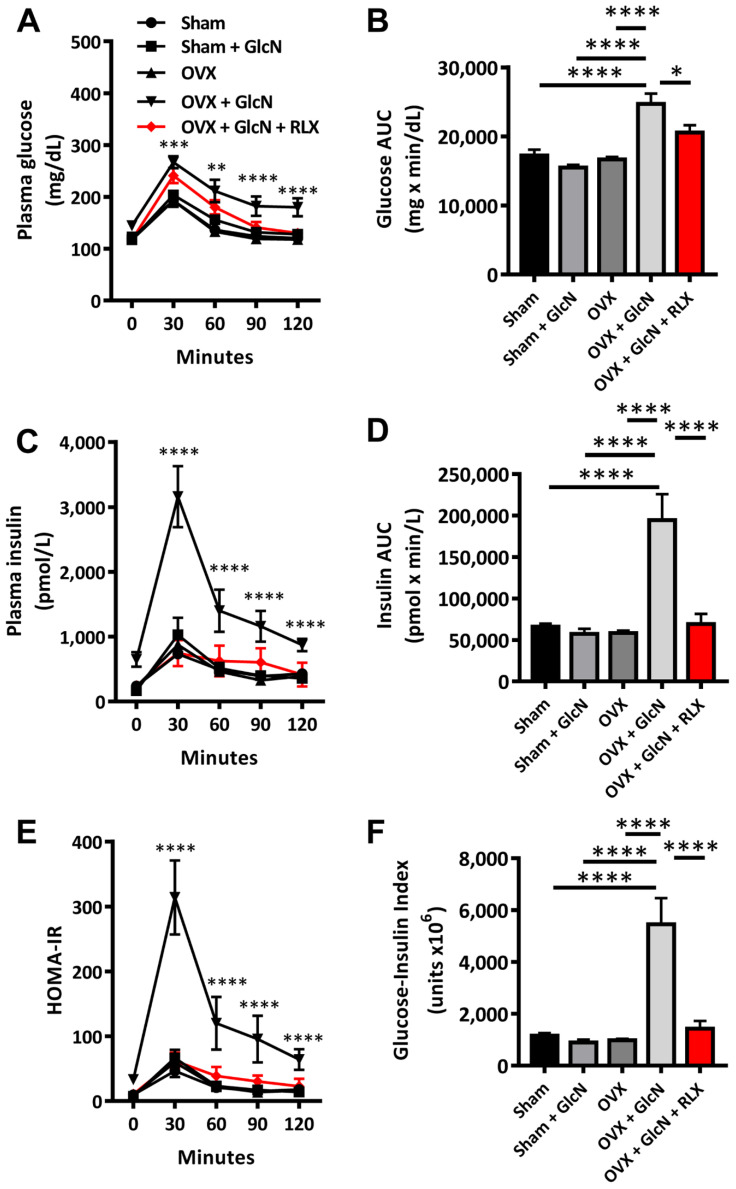
Levels and AUCs of plasma glucose, insulin, and HOMA-IR and glucose-insulin index in the IPGTT. The rats were randomly allocated to 5 treatments: (1) sham operation (Sham group), (2) sham with 750 mg/kg/d GlcN intraperitoneally (ip) injected for 14 days (Sham + GlcN group), (3) ovariectomy (OVX group), (4) ovariectomy with 750 mg/kg/d GlcN ip injected for 14 days (OVX+GlcN group), or (5) ovariectomy with 750 mg/kg/d GlcN ip injected for 14 days with subcutaneous RLX at 0.5 mg/kg/d. (*n* = 8 in each group). (**A**) The OVX + GlcN group exhibited elevated plasma glucose levels over all other groups at 30, 60, 90, and 120 min after glucose loading. Although the level of glucose in the OVX + GlcN + RLX group was higher than that in the other 4 groups, the difference did not reach statistical significance. (**B**) The OVX + GlcN group exhibited higher AUC for plasma glucose concentrations in the IPGTT than that in all other groups. RLX reduced the glucose AUC to nearly the level of the Sham group. (**C**) After glucose loading, the OVX + GlcN group demonstrated higher plasma insulin levels at 30, 60, 90, and 120 min than that in all other groups. (**D**) The OVX + GlcN group exhibited higher AUCs for plasma insulin concentrations than that in all other groups. RLX reduced the glucose-insulin index and the insulin AUC nearly to the levels in the Sham group. (**E**) Only the OVX + GlcN group exhibited higher HOMA-IR. The HOMA-IR of the 4 other groups exhibited no significant differences in the IPGTT. (**F**) The OVX + GlcN group demonstrated a higher glucose-insulin index. RLX reduced the glucose-insulin index to nearly the level in the Sham group. * *p* < 0.05; ** *p* < 0.01; *** *p* < 0.001; **** *p* < 0.0001.

The OVX + GlcN group exhibited elevated plasma glucose level (all *p* < 0.01) than that in all other groups at 30, 60, 90, and 120 min after glucose loading. Although the levels of glucose in the OVX + GlcN + RLX group were higher than those in the three other groups (Sham, Sham + GlcN, OVX), the difference did not reach statistical significance (Figure 2A). In addition, the OVX + GlcN group also exhibited significantly higher AUC for the plasma glucose concentrations in the IPGTT than that in all other groups (Figure 2B). After glucose loading, the OVX + GlcN group exhibited significantly higher plasma insulin levels at 30, 60, 90, and 120 min than that in all other groups (all *p* < 0.001; Figure 2C). The OVX + GlcN group demonstrated higher AUC for plasma insulin concentration (Figure 2D; *p* < 0.001) than that in all other groups. The OVX + GlcN group also exhibited elevated HOMA-IR (*p* < 0.001; Figure 2E). The glucose and insulin AUCs determine the glucose-insulin index. Only the OVX + GlcN group exhibited higher glucose-insulin index (*p* < 0.001; Figure 2F). RLX reduced HOMA-IR and the glucose-insulin index nearly to the levels in the Sham group, as determined from the IPGTT. In addition to reducing fasting glucose, insulin, and HOMA-IR, RLX significantly reduced plasma glucose, insulin levels, and HOMA-IR to nearly the levels in the Sham group, as determined from the IPGTT.

### 3.3. RLX Decreased Islet Size in the OVX + GlcN Rats

**Figure 3:**
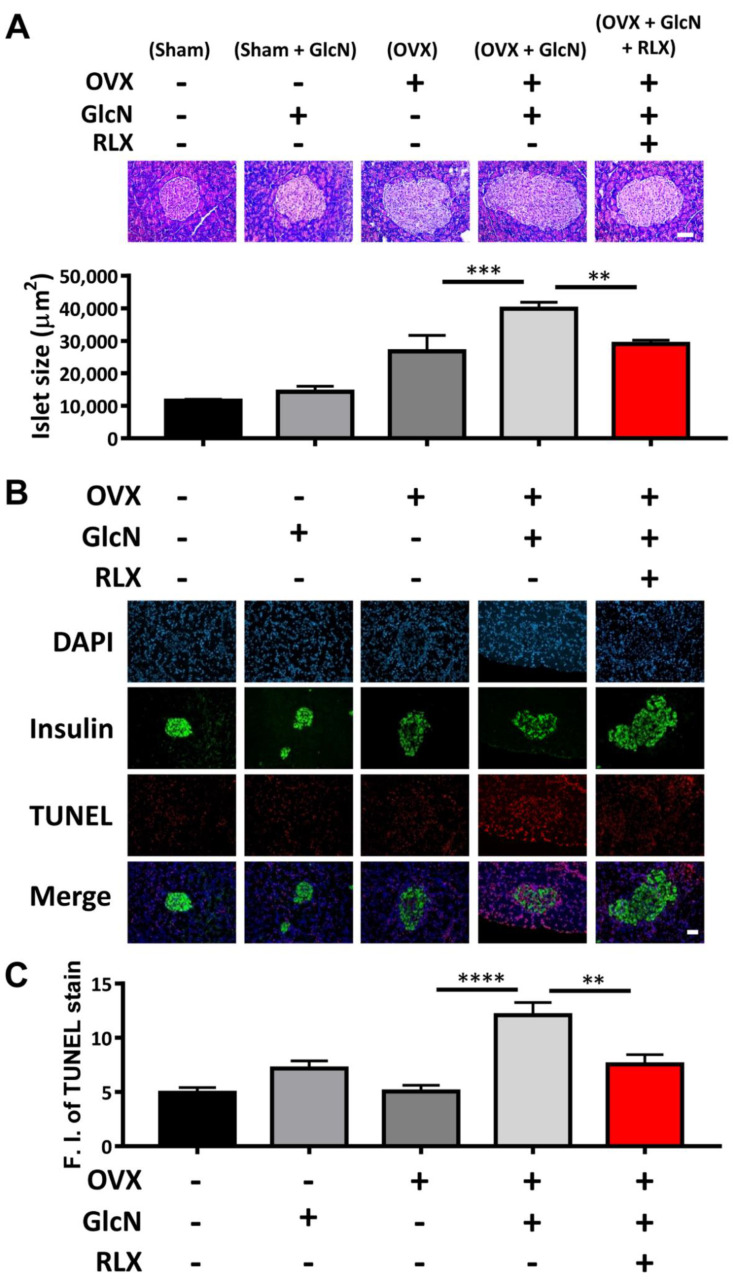
Islet size, immunofluorescence stains, and the quantification of insulin and TUNEL in rat pancreatic islets. The rats were randomly allocated to 5 treatments: (1) sham operation (Sham group), (2) sham with 750 mg/kg/d GlcN intraperitoneally (ip) injected for 14 days (Sham + GlcN group), (3) ovariectomy (OVX group), (4) ovariectomy with 750 mg/kg/d GlcN ip injected for 14 days (OVX+GlcN group), or (5) ovariectomy with 750 mg/kg/d GlcN ip injected for 14 days with subcutaneous RLX at 0.5 mg/kg/d. (*n* = 8 in each group). (**A**) The size of pancreatic islets of all groups. Pancreatic islets were nearly the same size in the Sham and Sham + GlcN groups. The size of the pancreatic islets markedly increased in the OVX group and increased further in the OVX+GlcN group, suggestive of islet hyperplasia compensating for IR. RLX treatment significantly reduced the size of the pancreatic islets in the OVX+GlcN rats to nearly the size in the OVX group. (Scale bar = 50 μm) (**B**) Immunofluorescence stains for TUNEL and insulin in pancreatic islets of all groups. Ovariectomy and GlcN reduced the immunofluorescence staining intensity for insulin in pancreatic islets; this intensity further decreased in the OVX+GlcN group. Treatment with RLX in OVX+GlcN rats increased the staining intensity of insulin. No difference was present among the OVX, GlcN, and Sham groups in apoptotic cell proportions as determined through TUNEL staining. (Scale bar = 50 μm) (**C**) Quantification of immunofluorescence stains for TUNEL in rat pancreatic islets. No difference existed among the OVX, GlcN, and Sham groups in the proportions of apoptotic cells. The proportion of apoptotic cells increased significantly in the OVX+GlcN group (12.09% ± 1.17%), whereas RLX reduced the proportion of apoptotic cells in the OVX+GlcN group (7.549% ± 0.90%). Each bar represents the mean ± SEM (*n* = 8–10 in each group). ** *p* < 0.01; *** *p* < 0.001; **** *p*  <  0.0001.

The pancreatic islets in the Sham and Sham + GlcN groups were nearly the same size. The size of the pancreatic islets in the OVX group enlarged significantly and further increased in the OVX + GlcN group; these results suggest that islet hyperplasia compensated for the initial IR (*p* < 0.001). RLX treatment decreased the pancreatic islets size in the OVX + GlcN rats to the size of those in the OVX group (*p* < 0.01; Figure 3A).

### 3.4. RLX Decreased Pancreatic Islet Apoptosis in TUNEL Stain in the OVX + GlcN Rats

Immunofluorescence staining for insulin as a marker of pancreatic islets and TUNEL staining to evaluate the level of apoptosis (Figure 3B) were performed. No difference was observed in apoptotic cells among the OVX, GlcN, and Sham groups in TUNEL staining. The proportion of apoptotic cells increased significantly in the OVX + GlcN group (12.09% ± 1.17%), whereas RLX reduced the number of apoptotic cells in the OVX + GlcN group (7.549% ± 0.90%; Figure 3C).

### 3.5. RLX Increased the Expression of PEPCK in the Liver and Decreased the Expression of GLUT-4 in the Soleus Muscle in the OVX + GlcN Rats

**Figure 4:**
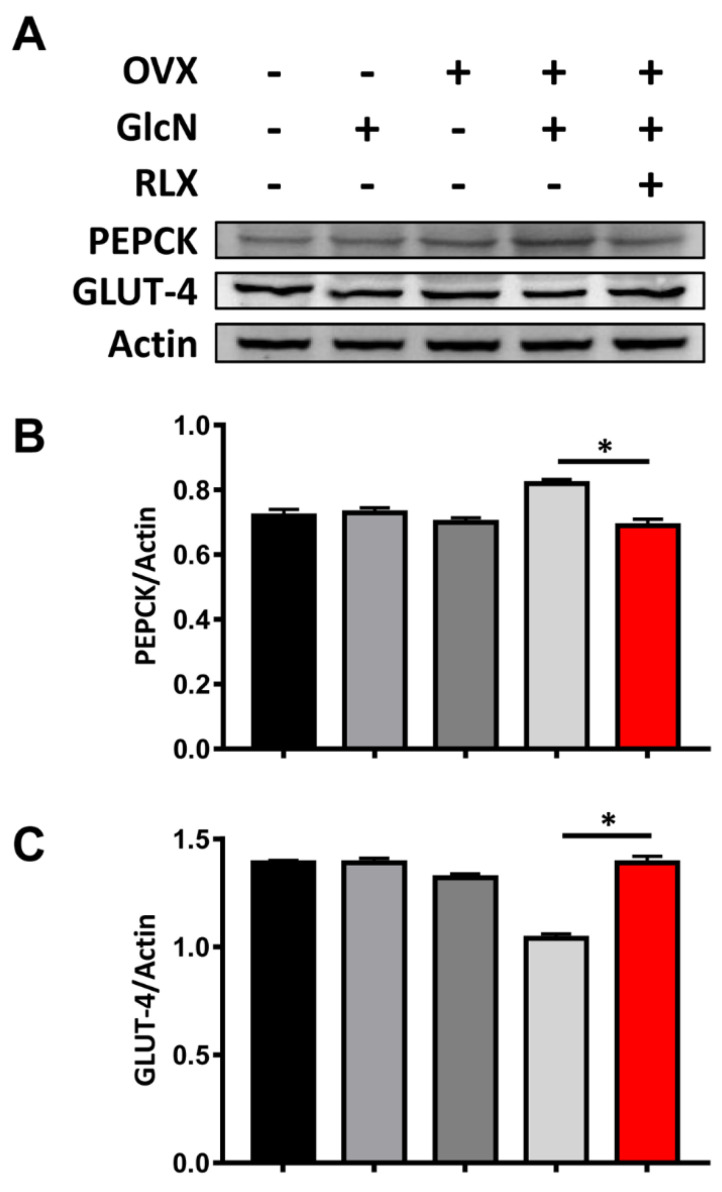
PEPCK expression in the liver and GLUT-4 expression in the skeletal muscle. The rats were randomly allocated to 5 treatments: (1) sham operation (Sham group), (2) sham with 750 mg/kg/d GlcN intraperitoneally (ip) injected for 14 days (Sham + GlcN group), (3) ovariectomy (OVX group), (4) ovariectomy with 750 mg/kg/d GlcN ip injected for 14 days (OVX+GlcN group), or (5) ovariectomy with 750 mg/kg/d GlcN ip injected for 14 days with subcutaneous RLX at 0.5 mg/kg/d. (*n* = 8 in each group). (**A**) Increased PEPCK expression in the liver and decreased GLUT-4 expression in soleus muscle were only observed in OVX rats given GlcN. RLX treatment reversed these effects. (**B**) The OVX + GlcN group exhibited increased PEPCK expression. RLX significantly reduced PEPCK expression in OVX rats with GlcN treatment to nearly the same level as the Sham group. (**C**) The OVX + GlcN group demonstrated decreased GLUT-4 expression. RLX preserved the expression of GLUT-4 in OVX rats with GlcN, maintaining it at nearly the level of the Sham group. No significant difference was present in the PEPCK or GLUT-4 expression among the Sham + GlcN, OVX, Sham, and OVX + GlcN + RLX groups. * *p* < 0.05.

OVX rats given GlcN presented with more PEPCK in the liver and less GLUT-4 expression in the soleus muscle. RLX treatment reversed these effects. No significant difference was observed in PEPCK or GLUT-4 expression among the Sham + GlcN, OVX, Sham, and OVX + GlcN + RLX groups (*p* < 0.05; Figure 4).

### 3.6. RXL Increased Extracellular Insulin Secretion and Cell Viability in MIN-6 Cells Treated with GlcN

**Figure 5:**
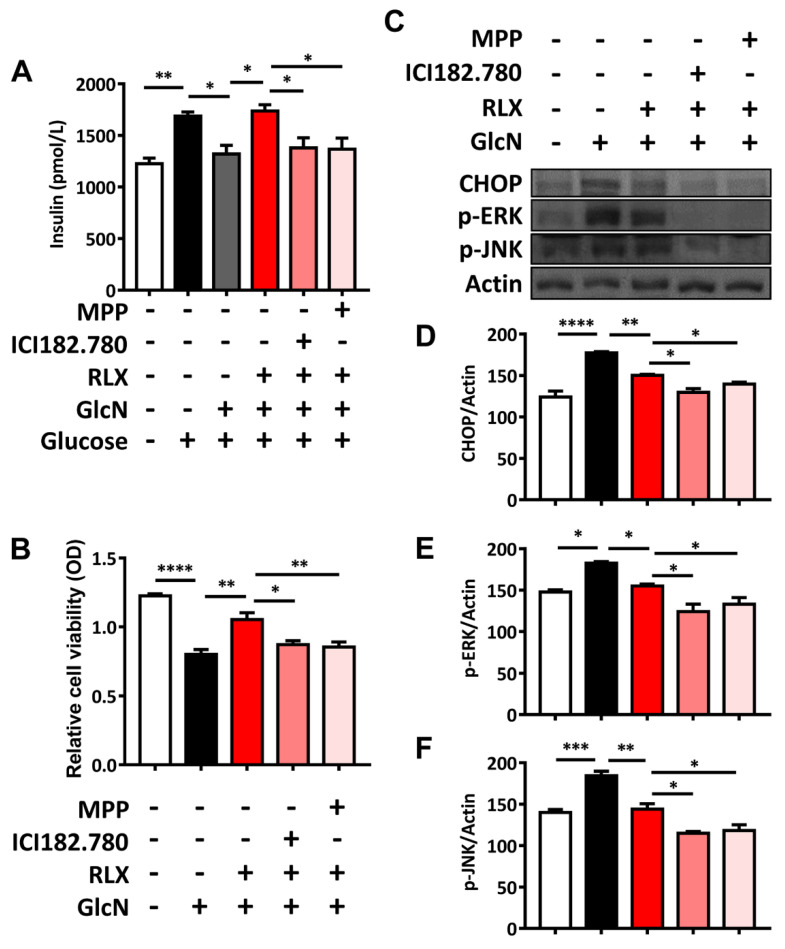
Extracellular insulin secretion, MTT assay, and Western blot analysis for the expression of ER stress-[associated proteins in MIN-6 cells. (**A**) Extracellular insulin secretion. Glucose increased extracellular insulin secretion, whereas GlcN reduced extracellular insulin secretion. RLX reversed GlcN-induced extracellular insulin secretion, whereas ICI 182,780 and MPP counteracted the effects of RLX. (**B**) MTT assay for cell viability. GlcN treatment reduced optical density in the MTT assay, whereas RLX counteracted this effect and even enhanced optical density. With combined ICI 182,780 and MMP treatment, the protective effect of RLX on cell viability was negated. (**C**) Western blot analysis for the expression of ER stress-associated proteins in MIN-6 cells: (**D**) CHOP, (**E**) p-ERK, and (**F**) p-JUK. GlcN treatment enhanced CHOP, p-ERK, and p-JNK expression, whereas RLX reversed these effects. Pretreatment with ICI 182,780 and MPP counteracted the reversal effect of RLX to protect against GlcN-induced CHOP, p-ERK, and p-JNK changes. Each bar represents the mean ± SEM. * *p* < 0.05; ** *p* < 0.01; *** *p* < 0.001; **** *p* < 0.0001.

In order to clarify the roles of estrogen receptor α and estrogen receptor β in the effects of RLX, the antagonists of estrogen receptor α and estrogen receptor β were used. ICI 182,780 (antagonist to both estrogen receptor α and estrogen receptor β) and MPP (antagonist specific to estrogen receptor α) were treated as indicated. Glucose increased extracellular insulin secretion, whereas GlcN reduced it. RLX reversed the GlcN-induced decrease in extracellular insulin secretion, whereas ICI 182,780 and MPP counteracted the effects of RLX on extracellular insulin secretion (Figure 5A). GlcN treatment reduced the optical density, as determined through the MTT assay, whereas RLX reversed this effect and increased optical density in the MIN-6 cells. Pretreatment with ICI 182,780 and MPP reduced the reversal effect of RLX, and the cell viability was similar to that after GlcN treatment (Figure 5B).

### 3.7. RXL Decreased the Expression of ER Stress-Associated Proteins CHOP, p-ERK, and p-JUN in MIN-6 Cells Treated with GlcN in Western Blot Analysis

GlcN treatment enhanced CHOP, p-ERK, and p-JNK expression, whereas RLX reversed these effects. Pretreatment with ICI 182,780 and MPP (estrogen receptor antagonists) counteracted the reversal effect of RLX to protect against GlcN-induced CHOP, p-ERK, and p-JNK increases (Figure 5C–F). These results indicate that GlcN induces ER stress, but RLX can relieve it. The effects of RLX are evident mainly through estrogen receptor α.

## 4. Discussion

In previous studies, we have concluded that GlcN-induced IR in OVX rats was related to reduced insulin secretion, pancreatic β-cell apoptosis, and enlarged pancreatic islets, and that E_2_ could counteract these adverse effects of in OVX rats [40,41]. In the present study, we further demonstrated that RLX, an SERM rather than an estrogen analog, can also ameliorate the deleterious effects of GlcN in OVX rats. Of the mechanisms underlying the protective effects of RLX which may be related to the combined effects of increasing GLUT-4 expression in skeletal muscle, decreasing PEPCK expression in the liver, inhibiting the growth of the pancreatic islets, and ameliorating ER stress in β-cells were shown to improve β-cell survival and function.

OP increases fracture risk and requires treatment to prevent subsequent comorbidities and mortality after fracture [19,22,25,26,49,50,51,52,53,54,55,56,57,58,59,60,61,62,63,64,65,66,67,68,69,70,71,72,73,74,75,76]. Knee OA leads to an increased risk of fall, which therefore increases fracture risk and required treatment [77,78,79]. OP and OA are common in older adults, especially in postmenopausal women, and they have negative impacts on quality of life [25,62,80,81]. OP is a clinically encountered comorbidity in older adults with OA [59,62,82,83]. Many patients (66%) with end-stage OA have OP or osteopenia [84]. Simultaneous lower extremity varus malalignment and OP in postmenopausal women result in more rapid OA development [85]. Hand OA and low hand and arm bone mineral density are related and can increase the risk of wrist fracture [86]. Therefore, the combined use of RLX and GlcN clinically is likely because of the frequent comorbidity of OA and OP in postmenopausal women. RLX ameliorated OA in an OVX rat model, indicating that it is a potential candidate for treating postmenopausal women with OA and OP [87]. RLX improved plasma fasting blood glucose levels in OVX rats with DM induced by a high-fat diet and ip administered streptozotocin [88]. In a clinical trial, RLX elevated 2 h insulin levels and the insulin AUC during the oral glucose tolerance test (OGTT) through reduced hepatic extraction. The insulin-retaining effect of RLX may be helpful in postmenopausal women with decreased insulin reserves or those predisposed to T2DM [89]. Compared with a placebo group, RLX resulted in significantly lower HOMA-IR levels in postmenopausal women with IR in a double-blind randomized trial [90]. RLX may improve glucose levels in postmenopausal women taking GlcN.

E_2_ reduces hepatic gluconeogenesis-related genes including phosphoenolpyruvate carboxykinase 1 (*Pck*-*1*) and glucose 6-phosphatase (*G6Pase*). The effects of E_2_ were inhibited in mice lacking liver estrogen receptor α. Hepatic estrogen receptor signaling is related to gluconeogenesis maintenance in men [91]. In the current study, RLX reduced liver PEPCK expression in OVX rats treated with GlcN. The effects may, at least partially, be exerted through liver estrogen receptor α.

Skeletal muscle estrogen receptor α plays a key protective role in the regulation of insulin action and metabolic homeostasis. Compared with estrogen receptor β and GPR30, estrogen receptor α is expressed much more in both rodent and human muscle [92,93]. Total muscular GLUT-4 level determined the IR phenotype in estrogen receptor α–knockout mice [94]. RLX can increase the mRNA expression of GLUT-4 in human skeletal muscle cells [95]. In the present study, RLX enhanced GLUT-4 expression in the soleus muscle. The effect of RLX on GLUT-4 may be through estrogen receptor α.

In a previous study, estrogen receptor α knockout reduced glucose-stimulated insulin secretion in a murine model. In addition, estrogen receptor α knockout in MIN-6 cells enhanced ER stress and apoptosis, and overexpression of estrogen receptor α reduced oxidative stress-induced CHOP expression in MIN-6 cells. Thus, estrogen receptor α targets β-cell apoptosis susceptibility and insulin secretion capacity [96]. We have previously reported that GlcN impairs the insulin secretion of β-cells and enhances β-cell apoptosis through increases in ER stress-related proteins including CHOP, p-ERK, p-EIF2α, and p-JNK in MIN-6 cells, whereas E_2_ ameliorates the ER stress caused by GlcN [41]. In the current study, RLX, similarly to E_2_, reversed these adverse effects of GlcN. RLX counteracted GlcN-mediated reductions in extracellular insulin secretion, cell viability, and the expression of ER stress-associated proteins. Both ICI 182,780 and MMP inhibited these benefits of RLX, with no statistical differences in extracellular insulin secretion or cell viability, but differences in the expression of ER stress-associated proteins. Our results indicate that RLX ameliorates GlcN-induced dysfunction in pancreatic β-cells through estrogen receptors, mainly estrogen receptor α for cell viability and extracellular insulin secretion, and from increases in ER stress-associated proteins. Further studies are required to explore the mechanism of RLX in ER stress-associated protein suppression.

There was one limitation to our study: we did not stain Ki67 to clarify the roles of proliferation in pancreatic β-cells. Further studies may be required to clarify the roles of proliferation in pancreatic β-cells.

## 5. Conclusions

GlcN may lead to β-cell dysfunction and apoptosis via diminishing pancreatic β-cell viability and insulin secretion and increasing ER stress-associated protein levels. RLX can reverse the effects of GlcN, but pretreatment with ICI 182,780 and MPP can inhibit the reversal effects of RLX. The effects of RLX are exerted mainly through estrogen receptor α. Uncovering more about the underlying mechanisms requires further study.

## Data Availability

Data are available on request.
